# Extreme Temperature and Mortality by Educational Attainment in Spain, 2012–2018

**DOI:** 10.1007/s10680-022-09641-4

**Published:** 2022-10-03

**Authors:** Risto Conte Keivabu

**Affiliations:** Department of Social and Political Sciences, European University Institute, Via della Badia dei Rocettini 9, 50014 San Domenico di Fiesole, Italy

**Keywords:** Mortality, Extreme temperature, Socioeconomic inequalities, Educational attainment, Climatic differences

## Abstract

Extreme temperatures are a threat to public health, increasing mortality in the affected population. Moreover, there is substantial research showing how age and gender shape vulnerabilities to this environmental risk. However, there is only limited knowledge on how socioeconomic status (SES), operationalized using educational attainment, stratifies the effect of extreme temperatures on mortality. Here, we address this link using Poisson regression and administrative data from 2012 to 2018 for 50 Spanish Provinces on individuals aged above 65 matched with meteorological data provided by the E-OBS dataset. In line with previous studies, results show that hot and cold days increase mortality. Results on the interaction between SES and extreme temperatures show a positive and significant effect of exposure to heat and cold for individuals with medium and low SES level. Conversely, for high SES individuals we do not find evidence of a robust association with heat or cold. We further investigate how the local climate moderates these associations. A warmer climate increases risks with exposures to low temperatures and vice versa for hot temperatures in the pooled sample. Moreover, we observe that results are mostly driven by low SES individuals being particularly vulnerable to heat in colder climates and cold in warmer climates. In conclusion, results highlight how educational attainment stratifies the effect of extreme temperatures and the relevance of the local climate in shaping risks of low SES individuals aged above 65.

## Introduction

Anthropogenic climate change raises new compelling questions on how the weather affects public health (Atwoli et al., 2021). For example, one of the outcomes of climate change is an increase in the occurrence of extreme temperatures and hot days (Fischer & Schär, 2010; Seneviratne et al., 2012) that have been linked with excess deaths in several climatic and economic contexts (Barreca et al., 2016; Carleton et al., 2022; Gasparrini & Armstrong, 2011; Gasparrini et al., 2015; Guo et al., 2018; Mora et al., 2017). Critically, some recent research on Europe highlighted how climate change could increase weather-related deaths (Forzieri et al., 2017) and reduce life expectancy in the future (Hauer & Santos-Lozada, 2021).

The literature on the association between temperature and mortality is large and growing (Basu, 2009). Extreme temperatures negatively affect the health of individuals forcing the body to react to maintain the internal temperature of comfort, but a failure to do so could be fatal (Cheshire, 2016). Most studies described the relationship between mortality and temperature as U-shaped (Gasparrini et al., 2015) and in some contexts as J-shaped (Chen et al., 2018a, 2018b) depending on the climatic area or the sociodemographic group analyzed. Consequently, some populations appear more vulnerable when exposed to extreme temperatures.

Important sociodemographic heterogeneities in risk related to temperature exist and have been highlighted in recent literature reviews (Kovats & Hajat, 2008; Son et al., 2019). The most important stratifier of risk is age, as individuals aged above 65 bear the highest toll of extreme temperatures due to a weaker cardiorespiratory system (Oudin et al., 2011). Similarly, women have been shown to be more vulnerable when exposed to extreme heat compared to men (Achebak et al., 2019). However, the evidence on the socioeconomic differences of risk to extreme temperatures such as determined by the educational level of individuals has been described as only suggestive by a recent literature review (Son et al., 2019).

In this article, we explore the educational gradient in temperature-related deaths posing two main questions: How does the educational attainment of an individual stratify the effect of extreme temperatures on mortality? How is the educational gradient of temperature-related deaths moderated by the local climate? In our analysis, we focus on the Spanish context leveraging administrative data provided by the Spanish Statistical Institution (Instituto Nacional de Estadistica – INE) on mortality by educational level for individuals aged above 65 from 2012 to 2018, in 50^1^ Spanish Provinces comprising approximately 2.5 million deaths. Moreover, we connect the administrative data on mortality with accurate meteorological information from the E-OBS gridded dataset.

This paper aims to provide further evidence on how socioeconomic status (SES) measured using the educational attainment of the deceased individual^2^ stratifies the relationship between temperature and mortality for individuals aged above 65, to a literature that has shown only limited or suggestive evidence of a higher burden for individuals with low educational attainment (Son et al., 2019). The existing research has shown low educated individuals to suffer the most exposure to extreme temperature in the European context (Borrell et al., 2006; Marí-Dell’Olmo et al., 2019) and in Jiangsu, China (Chen et al., 2016). However, some research has also shown the lack of an educational gradient in the USA (Isaksen et al., 2016). Consequently, we aim to provide new evidence to these contrasting findings on the educational gradient in extreme temperature exploring the Spanish territory that has been classified as hosting the highest risk of deaths attributable to heatwaves in Europe in the future (Forzieri et al., 2017).

Focusing on the educational gradient in mainland Spain, the Canary and Balearic Islands, we aim to improve on previous studies that inquired the SES gradient only in one specific context in the Spanish territory, Barcelona. An educational gradient in the exposure to heat for the population aged above 65 has been observed in Barcelona in the context of the heat wave of 2003 (Borrell et al., 2006), and a study inquired the educational gradient in the effect of extreme temperatures on mortality from 1992 to 2015 (Marí-Dell’Olmo et al., 2019). Both studies found all educational groups to be affected by extreme heat and cold but low educated individuals to suffer the most. However, these findings might not be generalizable to other contexts in Spain due to climatic differences that might alter how individuals respond to extreme heat or cold.

The local climate is a moderator of the effect of extreme climatic events on mortality. Individuals actively respond to the exposure to extreme weather events with technological and behavioral responses that limit risks in the future (Carleton & Hsiang, 2016; Kahn, 2016). Higher latitude and colder climates have been found to increase the risks related to extreme heat in the USA (Curriero et al., 2002; Medina-Ramón & Schwartz, 2007; Xiao et al., 2015), and to best of our knowledge, there is still no study documenting the same association for both heat and cold in Spain. In addition, we propose an additional interaction between the SES of individuals and the climatic characteristics of a geographical area. More precisely, we expect individuals aged above 65 with a lower SES to be the most affected by the local climate in their ability to adapt to extreme weather events, as they possess limited economic resources, and they are more dependent on local institutions to affront environmental risks.

The remainder of the article is organized as follows. In Sect. 2, we present the conceptual framework describing how extreme cold and heat are related to mortality, why we expect an educational gradient in this association and why the local climate could moderate this relationship. In Sect. 3, we give a description of the data, variables and empirical strategy used in the analysis. In Sect. 4, we report our main results. In Sect. 5, we present supplementary analysis and robustness checks. Finally, we conclude with a discussion in Sect. 6.

## Conceptual Framework

### Extreme Temperature and Mortality

Previous research on mortality and temperature documented an increase in deaths with exposure to cold and hot temperatures (Barreca et al., 2016; Dear & Wang, 2015; Díaz et al., 2018). Extreme temperatures determine medical conditions that compromise the effective functioning of the human cardiorespiratory system. In fact, some studies specifically studied excess mortality from cardiovascular and respiratory diseases in Spain (Achebak et al., 2019) and Changsha, China (Huang et al., 2014), or respiratory morbidity in Dongguan, China (Zhao et al., 2019). Nevertheless, the biological processes by which heat and cold affect human health are different.

The main cause of cold-related mortality is hypothermia. When exposed to cold temperatures, the body reacts with shivering to warm up or vasoconstriction to keep blood flow in the critical parts of the body (Osilla & Sharma, 2020). A failure in thermoregulation with exposure to extreme cold temperatures can cause severe medical conditions or death if prompt treatment is not given to the individual. More precisely, hypothermia happens when the body fails to keep temperature above 35 °C for a prolonged time (Cheshire, 2016). Moreover, cold days are often associated with mortality for a long period up to three weeks from the principal exposure, suggesting the effect of cold to persist over time (Bao et al., 2016; Huang et al., 2014). However, cold-related deaths are sometimes difficult to attribute to the direct exposure to cold and could be better attributed to indirect mechanisms (Arbuthnott et al., 2018). For example, exposure to moderate cold might trigger behavioral responses increasing the time spent indoors and help the spread of respiratory infections and that would increase the mortality rate.

Prolonged exposure to extreme heat is dangerous, as it might lead to hyperthermia. Usually, the body reacts to sweltering weather trying to cool down, sweating and losing fluids that need to be replaced by drinking water. Excessive loss of internal fluids and a failure in thermoregulation in a hot environment could finally result in heat stroke and death (Cui & Sinoway, 2014). Hyperthermia occurs when the body temperature fails to cool down and remains higher than 40 °C for an extended time (Cheshire, 2016). Compared to cold, the association between heat and mortality has shown to be limited in time with a lagged period of around 3 days suggesting a more intense short-term effect on individuals health that is easier to associate with the direct exposure (Huang et al., 2014).

A well-functioning cardiorespiratory system is critical with exposure to extreme temperatures, but some individuals could suffer from conditions leading to a comprised cardiorespiratory health and failed thermoregulation. Ageing is one of the main factors determining a weakened cardiorespiratory fitness (Jackson et al., 2009; Strait & Lakatta, 2012). In fact, research on temperature-related deaths documented a U-shaped relationship between temperature-related mortality and age (Bunker et al., 2016; Oudin et al., 2011). Infants are at risk as their cardiorespiratory system is not fully developed, increasing their likelihood of sudden infant death (Basagaña et al., 2011). Conversely, older individuals are more likely to have conditions affecting their cardio-respiratory system determining thermoregulatory deficiencies that can lead to death during prolonged exposure to extreme temperatures (Achebak et al., 2019).

Here, we described how extreme temperatures affect mortality and used age as an example of a factor leading to an enhanced risk of mortality and a relationship that is often described as U-shaped between temperature-related mortality and age. Next, we will discuss why we expect educational attainment to be a stratifier of the effect of temperature on mortality.

### Educational Attainment & Temperature-Related Deaths

Previous analysis exposed educational attainment to determine a heterogeneous effect of extreme temperatures on mortality. A recent literature review has found most of the studies showing a higher burden of extreme temperatures for individuals with a lower educational level (Son et al., 2019). For example, a higher risk of low SES individuals has been found in the European context (Borrell et al., 2006; Marí-Dell’Olmo et al., 2019) and in the USA (O’Neill et al., 2003; Zanobetti et al., 2013). However, some studies have also found no educational gradient in King County, Washington, USA (Isaksen et al., 2016), and Suzhou, China (Wang et al., 2014), posing questions on the generalizability of these studies to all geographical contexts.

The factors that would predict an educational gradient in temperature-related mortality are disparities in exposure, sensitivity, and social and medical support (Forastiere et al., 2007; Kovats & Hajat, 2008; Schifano et al., 2012). SES can reduce exposure to environmental risk as high SES individuals can purchase housing in a greener and fresher area (Gronlund, 2014).^3^ Moreover, heating (Chirakijja et al., 2019) and air-conditioning (Barreca et al., 2016) are important factors that can help to reduce exposure to extreme temperatures and might be more accessible to individuals with higher economic resources.

Also, disparities in vulnerability to extreme temperatures are likely determined by the educational gradient in health (Elo, 2009). Individuals with higher education are expected to live longer, healthier and be more resilient to exogenous stressors (Elo, 2009; Hummer & Hernandez, 2013). For example, individuals with low educational attainment are more likely to engage in unhealthy behaviors such as smoke, have eating disorders drink alcohol or develop health conditions such as cardiovascular disease, obesity and diabetes (Link & Phelan, 1995; Son et al., 2019). These health conditions and behaviors are linked to impaired thermoregulatory function determining lower resilience to extreme temperatures (Cui & Sinoway, 2014). Additionally, an educational gradient has been observed in the seasonal pattern of mortality suggesting low SES individuals to be less resilient to environmental stressors (Rau, 2007).

Social support and paid elderly care have been found to be relevant in limiting temperature-related deaths (Klinenberg, 2002; Schifano et al., 2012). For example, a program of social support in Italy has shown to considerably reduce the likelihood of dying during the summer for older individuals (Schifano et al., 2012). In this regard, high SES individuals could be more likely to have access to social support, paid elderly care and medical institutions providing them with critical help during severe heat spells (Wang et al., 2021).

Based on the larger evidence suggesting higher mortality with extreme temperatures for low SES individuals and the described disparities in exposure, sensitivity and social support we expect low educated individuals to suffer the most when exposed to extreme temperatures in the Spanish context.

### Spain, One Country, Many Climates, Different Adaptation?

**Fig. 1:**
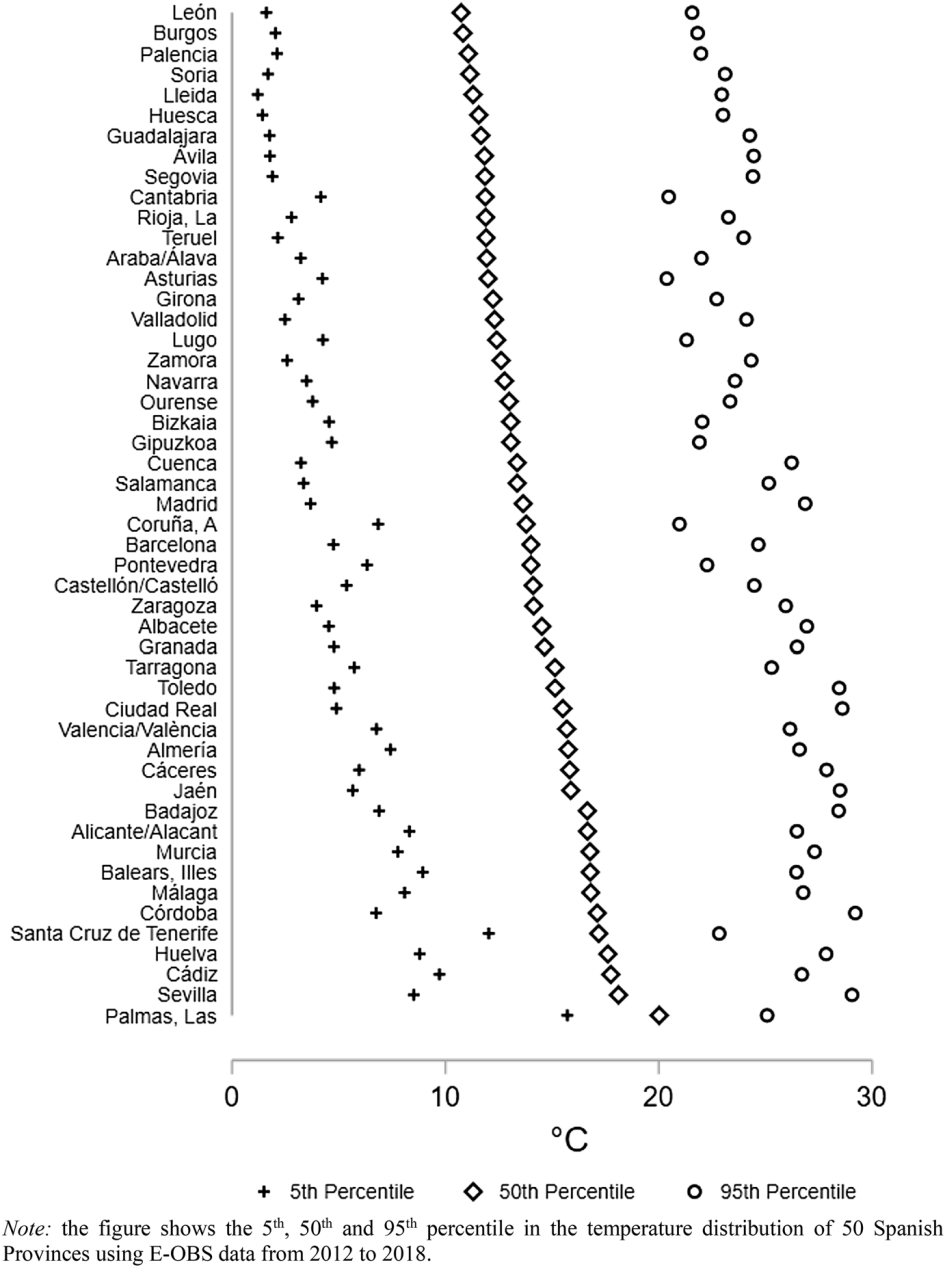
Climatic differences in 50 Spanish Provinces

Spain is a Mediterranean country and hosts a wide array of climates that might shape individual’s preparedness to extreme temperatures. For example, in Fig. 1 is depicted the 5th, 50th and 95th percentile of the daily average temperature distribution in the period 2012 to 2018 in 50 Spanish Provinces exposing large variation in the territory. The highest median temperature is recorded in Las Palmas at around 20 °C and the lowest in Lèon at around 10.7 °C. The 95th percentile of the temperature distribution ranges from 20.4 °C in Asturias to 29.2 °C in Cordoba and the 5th percentile from around 1.2 °C in Lleida to 15.7 °C in Las Palmas.

Temperature-related deaths have been decreasing over time in Spain (Achebak et al., 2019; Díaz et al., 2018; Follos et al., 2021) suggesting that individuals are able to adapt to extreme weather events (Kahn, 2016). For example, in the US context, the availability of cheaper heating (Chirakijja et al., 2019) and access to air-conditioning (Barreca et al., 2015, 2016) have helped reduce temperature-related deaths over time. In Spain, there is evidence that the implementation of housing policies helping the most vulnerable has reduced cold-related deaths (Marí-Dell’Olmo et al., 2017). However, the presence of air conditioning appears to not be associated with a reduction in heat-related mortality over time in Spain (Díaz et al., 2018), but a public policy has been shown to be effective in decreasing mortality determined by heatwaves (Martínez-Solanas & Basagaña, 2019). All in all, there is still no clear evidence of which factors were the most relevant in reducing temperature-related deaths in Spain over time (Achebak et al., 2019). Nevertheless, heterogeneities between climatic regions in the preparedness to cope with extreme temperatures might still exist.

Differences in climatic conditions have been shown to influence the preparedness of the local population to affront extreme temperatures affecting the shape of the association between temperature and mortality. For example, studies have either documented a U-shaped (Scovronick et al., 2018) or a J-shaped (Chen et al., 2018a, 2018b) relationship between temperature and mortality that is also dependent on the climatic context of analysis (Bao et al., 2016). In the US context, studies have highlighted how individuals living in colder climatic regions suffer more from the exposure to high temperatures and vice versa for individuals living in warmer areas (Curriero et al., 2002; Xiao et al., 2015). A possible explanation is that individuals living in colder climates are less adapted to extreme temperatures, lacking technologies, housing facilities or public policies that help to protect them from sweltering weather (Curriero et al., 2002). Moreover, we can expect low SES individuals to be more likely than high SES individuals to be vulnerable to heat in cold climates and cold in warm climates, in line with studies highlighting a larger burden of health shocks on poor individuals (Leonard et al., 2017). For example, living costs to affront both high and low temperatures would imply a larger economic burden on the household, exposing low SES individuals to additional expenses that could not be met and increase mortality (Chirakijja et al., 2019). Similarly, low SES individuals are more reliant on local institutions and public policies (Leonard et al., 2017) that might be less developed in colder regions to affront heat events or to face cold spells in warm climates.

Finally, we expect the high variation in Spanish temperatures to determine heterogeneous associations between heat, cold and mortality depending on the local climate and mostly so for low SES individuals.

## Dataset, Variables and Empirical Strategy

### Data

For the analysis, we used Spanish register data on all causes of death on individuals aged above 65 from 2012 to 2018 provided by the Spanish National Statistics (Instituto Nacional de Estadistica—INE). Unfortunately, information on the educational attainment of deceased individuals is often not available in the national administrative records, but there are some exceptions. In our case, we leveraged data for Spain that comprises information on the sociodemographic characteristics, educational attainment and the causes of death codified using the “International Classification of Death” (ICD) 10 at the individual level. The period of analysis is limited from 2012 to 2018 as the information on the educational attainment for the whole Spanish population is not available in the preceding years. This information has been computed using an algorithm and made available in 2016 (INE, 2016). Moreover, the data have been shown to be reliable and used in previous studies looking at the educational gradient in longevity and life span variation in Spain (Permanyer et al., 2018). Nevertheless, ours is the first study that uses this dataset to inquire temperature-related deaths. In fact, previous studies on the educational gradient in Spain focused only on the city of Barcelona using local administrative data (Borrell et al., 2006; Marí-Dell’Olmo et al., 2019). We classified individuals into three main educational groups “Low SES,” “Middle SES” and “High SES” individuals. Low SES individuals are those that did not complete elementary education; Middle SES those with completed elementary education; High SES those that have attended more than elementary education. We chose this classification to allow comparability with previous studies (Marí-Dell’Olmo et al., 2019).^4^ Overall, the total number of individual deaths amounts to 2,416,503^5^ and occurred in 50 Spanish provincial capitals in the population aged above 65 for all causes of deaths from 2012 to 2018.

We computed population exposures by educational group combining two sources of data. First, we gathered aggregate population estimates by province, year, age group and gender provided by INE. However, this information does not provide statistics by educational background. Consequently, we constructed estimates of educational attainment by gender, province and year for individuals aged above 65 using survey data provided by the Spanish Labor Force Survey (Encuesta de Poblacion Activa—EPA). The EPA is a national survey that gathers information every trimester on the Spanish population’s labor force characteristics. We used this data to capture the average proportion of individuals aged above 65 by educational level in each year, province and gender category and combined it with the population counts to calculate the population exposures by each sociodemographic group, province and year. Nevertheless, using survey data to calculate population estimates at the provincial level could determine some biases. To test the accuracy of our estimates, we compared the proportion of individuals in the three SES groups with those provided by INE using the highest administrative level available. More precisely, we used information on the percentage of individuals aged above 16 residing in 17 Spanish regions (Comunidades Autonomas) and compared them with our estimates based on the EPA microdata for the same population and administrative unit. Comparing these two measures, we observe very similar population statistics suggesting a low level of bias in our estimates (Appendix: Table 2).

The meteorological data are provided by the E-OBS and comprises complete and reliable daily data on all Spanish Provinces collected by local meteorological stations and interpolated to the whole Spanish territory (Cornes et al., 2018). The gridded dataset is at a 0.1° resolution, and we calculated the average values of all the grids falling within each provincial administrative boundaries to create a single daily meteorological observation for each province, as previously done in other studies (Achebak et al., 2019, 2020). Using this procedure, we collected information on our main variable of exposure mean temperature and on the control variables precipitation, solar radiation, wind speed and relative humidity.

We added to our analysis information on air pollution, gross domestic product (GDP) and population density. To do so, we gathered data on air pollution using the Copernicus Atmosphere Data Store (CAMS) that offers information on air quality for the whole European territory, comprising Spain. The dataset is provided on air pollutants such as PM10, PM2.5 and Ozone on a geographical resolution of around 0.75° × 0.75° and is gathered using satellite observations. An alternative source of data on air pollution collected by local measurement stations is available from the Spanish “Ministry of the Ecological Transition”^6^ measuring the levels of air pollutants such as particulate matter 10 (PM10), PM2.5, nitrogen dioxide (NO2) or ozone (O3). However, these stations are not widely present in the whole provincial territory and have several missing values in the daily estimates determined by discontinuous measurement that would highly bias or reduce the sample used in the main analysis. Moreover, we collected data on yearly values of GDP in current market prices in each province from 2012 to 2018 using data made available by INE. Finally, for population density we used the same information we collected to construct mortality rates provided by INE, but in this case we gathered data on the total population residing in the province in each year.

### Variables

We aggregated the individual deaths for the population aged above 65 by the specific sociodemographic groups within the provinces observed monthly from January 2012 to December 2018. More precisely, the total number of observations registering the sum of individual deaths is 25,200 and corresponds to the monthly time series of two gender categories, three SES groups in 50 provinces, observed over 7 years. Similarly, to construct the population exposures for individuals aged above 65, we used the yearly population estimates by province, gender and SES divided by 12 months and used linear interpolation between years to retrieve the monthly values.

We connected the mortality data with precise meteorological observations on the temperature of each province using the E-OBS dataset. We used the daily average temperature registered in each province to construct monthly temperature bins using percentiles of the province specific temperature distribution. More precisely, we constructed 7 temperature bins capturing the number of days in which the provincial temperature is below and equal to the 5th percentile; from the 5th to the 10th percentile; from the 10th to the 25th percentile; above the 25th and below the 75th percentile; from the 75th to the 90th percentile; from the 90th to the 95th percentile; above the 95th percentile. The category with temperatures between the 25th and the 75th percentile is considered at the comfort zone and dropped from the analysis. The temperature exposures have been constructed in this way for three main reasons. First, using monthly temperature bins we can capture non-linearity in the relationship between temperature and mortality that, based on previous studies, has been described as U-shaped or J-shaped (Barreca et al., 2016). Secondly, using monthly temperature exposures allows to capture lagged responses to high temperatures (Barreca et al., 2016; Hovdahl, 2020). Most research using daily data models the lag response adding lags that vary from 7 to 21 days (Vicedo-Cabrera et al., 2021). Thirdly, we use the percentiles in the temperature distribution within each province to capture location specific extreme temperatures permitting to account for different temperature–mortality response functions in each province that might vary due to differences in the adaptation to the local temperature (Vicedo-Cabrera et al., 2021).

We add five control variables, gender, SES, air pollution, GDP per capita, population density. We operationalized air pollution as monthly average exposure to particulate matter 2.5 (PM2.5) in the province. We used PM2.5 as it is one of the most harmful pollutants widely used in the literature (Colmer et al., 2020). For GDP per capita we used the values on GDP provided by INE and computed the GDP per capita using the total population in each province retrieved by the population estimates used to construct the exposures. Moreover, to create monthly values for GDP per capita we used linear interpolation between years. Finally, we computed population density using the total yearly population^7^ in each province and divided it by the provincial area^8^ calculated in square kilometers and used linear interpolation between years to construct monthly values.

### Empirical Strategy

To estimate the effect of temperature on deaths in the population of individuals aged above 65, we use Poisson regression models with logarithmic link, offsets as exposures and monthly covariates. The equation can be described as the following:

1 $$\log (Y_{pymgs} ) = \log (E_{pymgs} ) + \sum_{j} \theta_{j} TEMP_{pym} + \beta X_{pym} + \alpha_{pm} + \delta_{ym} + e_{pymgs}$$

In Eq. 1, *Y*_*pymgs*_ denotes the count of deaths in province *p*, month *m,* year *y,* gender group *g* and SES group *s. E*_*pymgs*_ is introduced as an offset term capturing the exposure to the risk of death in each province, year, month, gender and SES group. $TEMP{j}_{pym}$ represents the monthly temperature bins of the province specific percentiles in the temperature distribution that are ≤ 5th; > 5th and ≤ 10th; > 10th and ≤ 25th; ≥ 75th and < 90th; ≥ 90th and < 95th; and ≥ 95th. The days with temperatures falling within the 25th and 75th percentile is set at the reference level and considered at the comfort zone.

Compared to previous studies using Ordinary Least Squares (OLS) approach with the logarithm of the monthly mortality rate as the outcome variable (Barreca et al., 2016; Hovdahl, 2020), we use Poisson regression and an offset term for the population exposures for the advantages it presents when modeling count data (O’Hara & Kotze, 2010). Conversely, the fixed effect setting we adopt is widely used in the literature on temperature-related mortality and relies on the assumptions that no other time-varying factors correlate both with the variable of exposure and the outcome (Barreca et al., 2016; Hsiang, 2016). We introduce ${\alpha }_{pm}$ to capture province-by-month fixed effects and ${\delta }_{my}$ to capture month-by-year fixed effects and cluster standard errors at the province level to correct for autocorrelation within provinces over time (Cameron & Miller, 2015). The fixed effects are used to control for possible time-varying factors and seasonal trends that might correlate with temperature and mortality across every province and observed over time (Barreca et al., 2016). Consequently, we expect the random variation in weather conditions from the monthly province mean between years to explain the variation in the monthly mortality rate determined by the temperature exposure. Nonetheless, we include a vector of control variables to capture the effect of possible confounding factors that in our case are precipitation, relative humidity, solar radiation, wind speed, air pollution, GDP per capita, population density, SES (Low SES; Medium SES; High SES) and gender (female = 1). For example, air pollution has been shown to modify the effect of temperature on mortality (Chen et al., 2018a, 2018b), but in previous studies on Spain this has not been considered for lack of comprehensive data (Borrell et al., 2006; Marí-Dell’Olmo et al., 2019).

Finally, to estimate the stratified effect of extreme temperatures by the SES groups, we estimate Eq. (1) adding an interaction between the temperature bins and the SES groups. Moreover, to test how the local climate moderates the effect of heat and cold on mortality we add another interaction with the median temperature in the province.

## Results

In Table 1, we present summary statistics of the monthly, provincial and sociodemographic group averages for the main variables from 2012 to 2018. First, we can observe the distribution of days within the 7 temperature bins. As expected, the highest average number of days is observed at the comfort zone (25th to 75th percentile). Interestingly, we find a higher average and maximum number of days in the hottest bin with temperatures > 95th percentile compared to temperatures between the 90th and 95th percentile. These statistics are determined by the year 2015 that has been one of the hottest recorded in Spain and Europe (Russo et al., 2015). In fact, excluding the year 2015, the average number of days in the hottest temperature bin is reduced to 1.44 and the maximum number of days to 18. Additionally, we present the monthly average death counts and population exposures of individuals aged above 65 for the pooled sample and by SES group in the 50 provinces. In the control variables is notable the maximum value of 71.01 µg/m3 for PM2.5 that is found in the province of Ourense in October 2017 that saw a major wildfire affect this area and critically reduce air quality. Moreover, as noted in the previous section, the total number of observations adds to 25,200 comprising each gender and SES category observed monthly over 7 years in each of the 50 provinces.

**Table 1:** Summary statistics of the monthly province sociodemographic group observations

Variable	Mean	Std. Dev.	Min	Max
*Temperature variables*				
≤ 5th percentile	1.62	3.44	0	23
> 5th and ≤ 10th percentile	1.58	2.56	0	19
> 10th and ≤ 25th percentile	4.63	5.68	0	23
> 25th and < 75th	14.95	9.86	0	31
≥ 75th and < 90th percentile	4.48	6.27	0	28
≥ 90th and < 95th percentile	1.56	3.02	0	17
≥ 95th percentile	1.63	3.73	0	29
*Death counts*				
Sum of deaths	95.89	113.16	0	1,187
Low SES sum of deaths	93.66	98.15	0	970
Medium SES sum of deaths	106.49	117.25	10	1,187
High SES sum of deaths	87.52	121.88	2	1,048
*Population exposures*				
Population exposures 65+	2,384.71	3,073.35	52.47	29,357.38
Population exposures Low SES 65+	1,987.42	2,155.39	52.47	14,051.42
Population exposures Medium SES 65+	2,706.31	3,252.03	398.46	22,043.77
Population exposures High SES 65+	2,460.38	3,584.73	130.05	29,357.38
*Control variables*				
GDP per capita (per 1,000)	22.16	4.78	14.44	37.1
Population density	129.64	167.81	8.57	827.23
Solar radiation	1017.51	4.49	1004.72	1034.48
Wind speed	0.46	1.02	−4.58	4.89
Precipitation	1.48	1.65	0	11.44
Relative humidity	68.08	12.55	26.72	95.04
PM2.5	9.96	3.87	3.15	71.01
*Observations*				
No. Province-year-month-sociodemographic group	25,200			

Summary statistics are calculated as averages for each monthly time series of observations for each gender and SES group in the 50 provinces from 2012 to 2018. Moreover, we show the population exposures and sum of deaths separately for the three SES groups

**Fig. 2:**
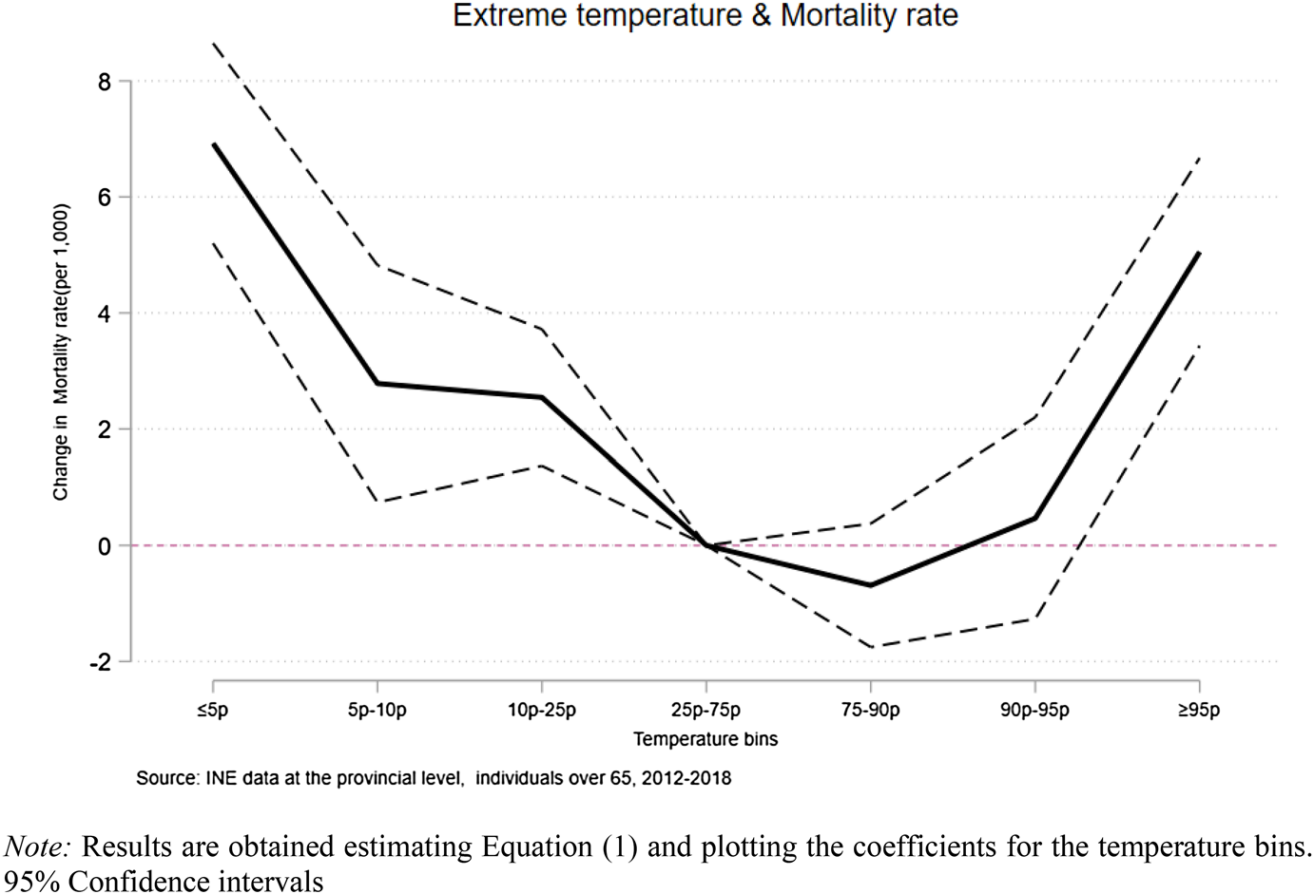
Extreme temperature and mortality rate

In Fig. 2 and in Appendix: Table 3, we present the results of the analysis on mortality and temperature for the pooled sample. The results for the pooled sample show a U-shaped relationship between temperature and mortality for individuals aged above 65. Temperatures lower and higher than the comfort zone show to increase mortality with a larger effect size at the most extreme bins. For cold days, we observe a larger effect size when temperatures are below or equal to the 5th percentile representing an increase in the monthly mortality rate of about 7 per 1,000 and for hot days of about 5 per 1,000 when these are above or equal to the 95th percentile, respectively.

**Fig. 3:**
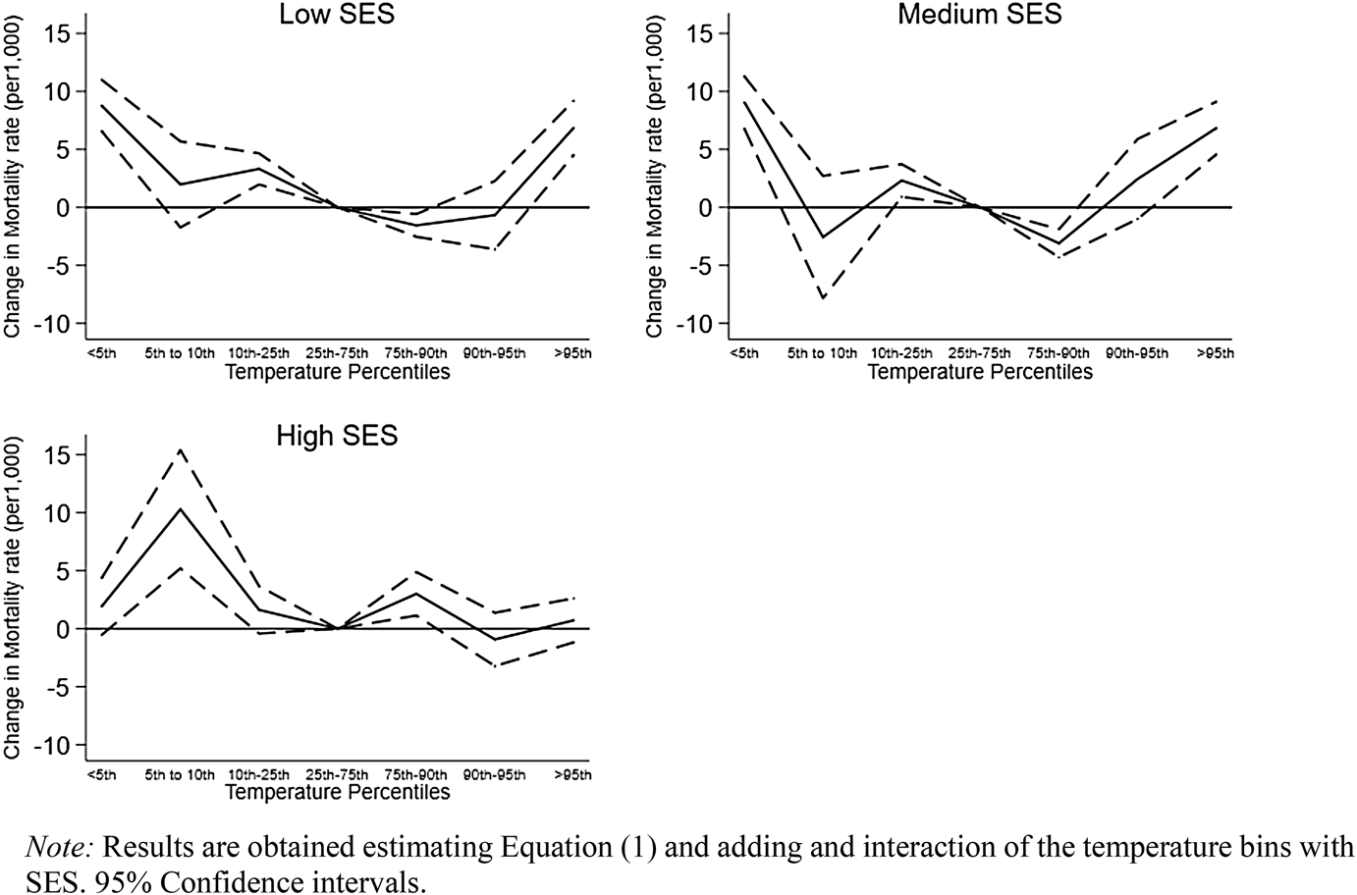
Extreme temperature and mortality rate by SES groups

In Fig. 3 and in Appendix: Table 4, we plot the results of the interaction between the temperature bins and SES. Interestingly, we observe a U-shaped relationship between temperature and mortality for Low and Medium SES individuals. Conversely, the association shows to be flatter for High SES individuals. Days with temperature ≤ 5th percentile increase mortality in Medium and Low SES groups, but slightly more for the latter. An extra day with temperatures ≤ 5th percentile compared to a day in the comfort zone increases the monthly mortality rate by 6 per 1,000 in the pooled sample, by 8 per 1,000 for Low and Medium SES individuals. Conversely, for High SES individuals the coefficient is smaller with a value of 1 per 1,000 and fails to reach statistical significance. For hot days, the SES gradient is like what observed for cold days. An increase in deaths with exposure to hot days is evident both for Low and Medium SES individuals. More precisely, we observe days above or equal the 95th percentile to increase the monthly mortality rate for Low and Medium SES individuals by 8 per 1,000, and for High SES individuals by 2 per 1,000. An interesting pattern is observed for moderate temperatures in the ranges ≥ 5th to 10th percentile and ≥ 75th to 90th as Medium and Low SES individuals show a negative value for the interaction suggesting lower risk. Possibly, the pattern could be linked to indirect mechanisms (Arbuthnott et al., 2018).

Overall, the results highlight both cold and hot days to increase the mortality rate for Low and Medium SES individuals. Conversely, High SES individuals appear to be less affected by the negative effects of extreme temperatures.

**Fig. 4:**
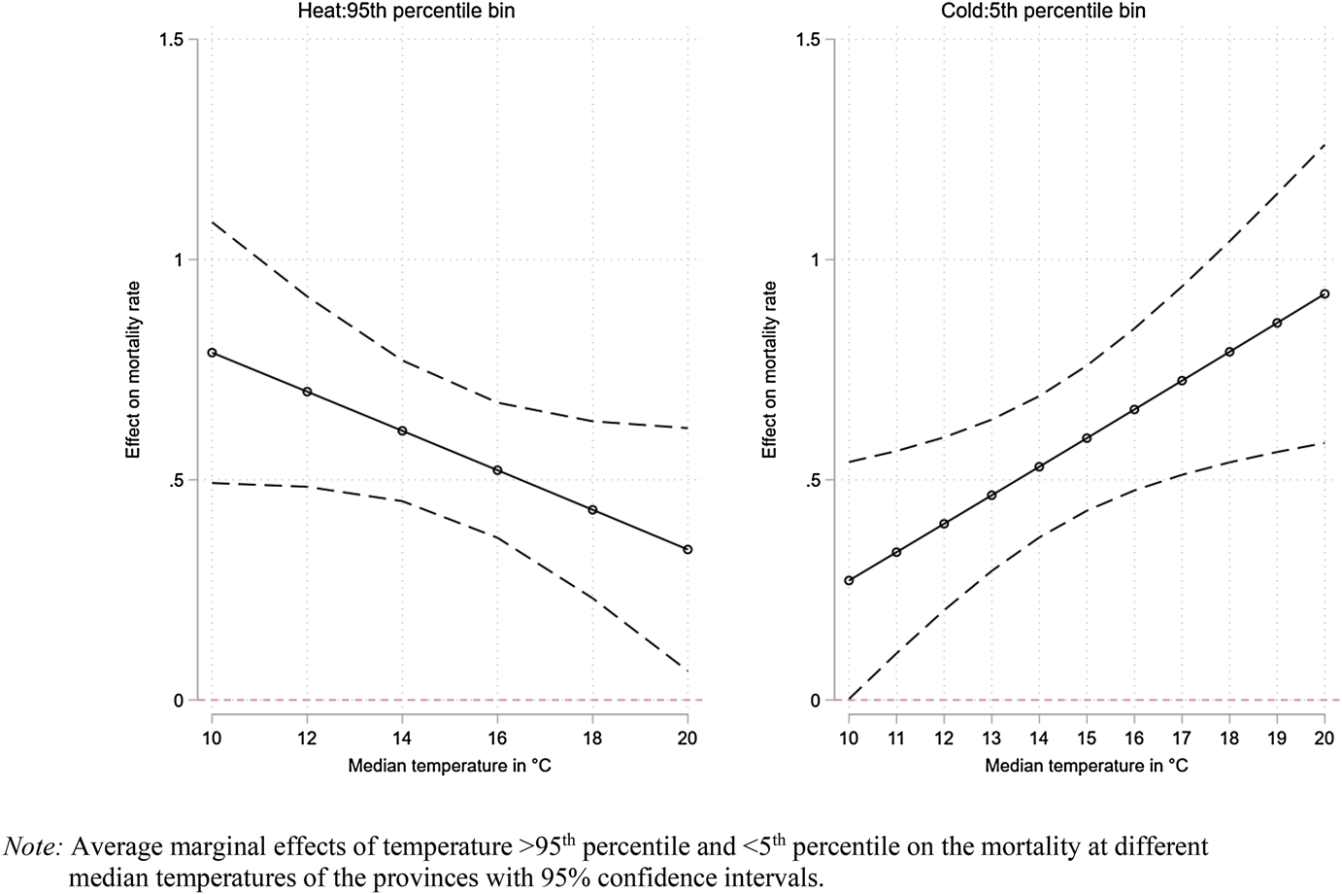
Hot and cold days & mortality rate by median temperature

In Fig. 4 (Appendix: Table 5), we further inquire how the relationship between temperature and mortality varies depending on the provincial median temperature adding an interaction between the temperature bins and the median temperature. The interaction term between the median temperature and days colder than the comfort zone mostly shows a positive coefficient, suggesting individuals residing in warmer climates to be more susceptible to cold temperatures. In fact, focusing on the most extreme bin we can observe a negative relationship suggesting an increase in cold-related deaths in warmer climates. Conversely, the interaction term with days warmer than the comfort zone mostly shows a negative coefficient suggesting that provinces with a warmer climate experience a lower risk when exposed to hot days.

**Fig. 5:**
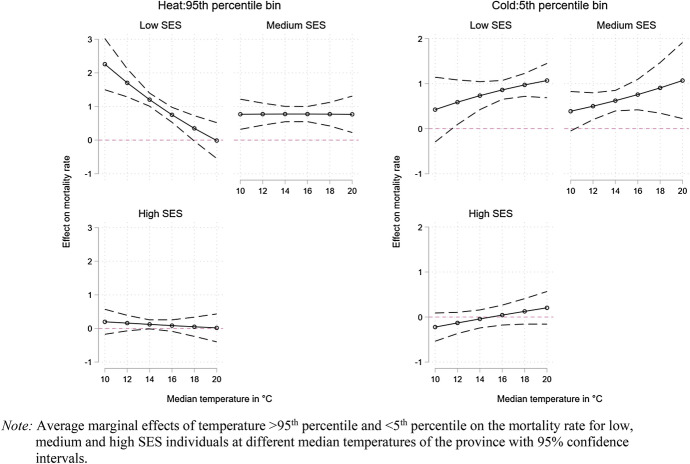
Mortality, hot and cold days by median temperature and SES

In Fig. 5 (Appendix: Table 6), we add a third interaction with SES to explore how the median temperature influences temperature-related mortality for the three SES groups. Here, we observe an increase in cold-related deaths for all SES groups with a higher median temperature. For hot days, the SES differences are noticeable as the association is flat for Medium and High SES individuals but appears negative for Low SES individuals. More precisely, for Low SES individuals the effect of days with temperatures above or equal the 95th percentile shows a constant decline with a higher median temperature suggesting Low SES individuals to be ill prepared to affront hot days in colder climates.

## Supplementary Analysis & Robustness Checks

We further investigate factors of risk associated with extreme temperatures exploring how gender, marital status and age moderate the effects of temperature on mortality. First, we run the analysis adding an interaction between gender using the binary variable (Women = 1) provided both in the death registers and population estimates and that we use to construct aggregate death counts and exposures for these separate sociodemographic groups. The results by gender (Appendix: Table 7) expose a stronger effect of heat and especially cold for women. The results for hot days mirror the results of previous studies documenting a larger burden of heat for women in Spain (Achebak et al., 2019; Marí-Dell’Olmo et al., 2019). However, we do not observe a larger burden of cold for men, as shown in previous studies (Achebak et al., 2019; Marí-Dell’Olmo et al., 2019). We further explored the interaction between temperature bins and gender separately by SES groups (Appendix: Table 8). A higher educational attainment reduces risks with exposure to cold and heat for both genders. High SES women and men show coefficients with low statistical power and size compared to Medium and Low SES individuals of both genders showing a stronger effect size when exposed to extreme heat and cold. The results for women confirm what has been found in previous studies, that educational attainment effectively reduces their risk when exposed to extreme temperatures (Marí-Dell’Olmo et al., 2019). Conversely, the findings for men are slightly different from what found in (Marí-Dell’Olmo et al., 2019) where men without studies were less affected by cold compared with those with primary education or more.

There is some suggestive evidence that married individuals are less vulnerable to exposure to extreme temperatures as they can support each other during days of uncomfortable weather. We created a dummy variable (Married = 1) and interacted it with the temperature bins to inquire the existence of heterogeneous effects based on marital status (Appendix: Table 9). As expected, we found a larger effect size of extreme temperatures for not married individuals. We further inquired differences by marital status and SES (Appendix: Table 10) observing a larger effect size for cold and heat in not married low SES individuals. Conversely, the risk of temperature exposure for High SES individuals is less dependent on their marital status.

**Fig. 6:**
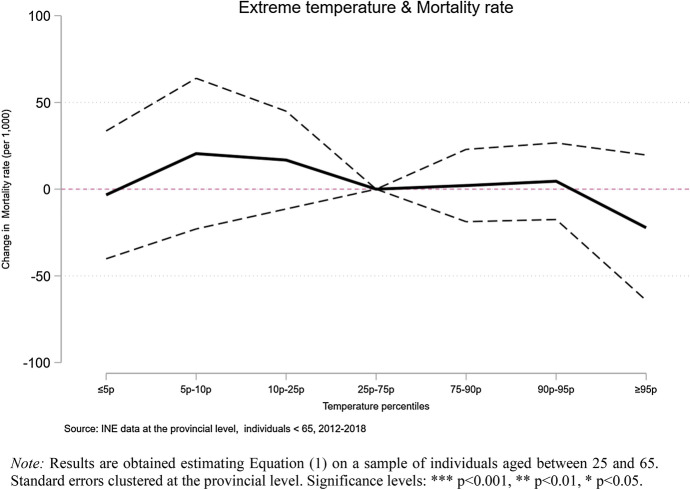
Extreme temperatures and mortality individuals aged below 65

In our analysis, we decided to focus on individuals aged above 65, as extensive studies have shown they are the most affected by extreme temperatures. We replicated the analysis in Fig. 2 with individuals aged 25 to 64 (Appendix: Fig. 6). Here, we do not observe any substantive findings. We run the same analysis adding an interaction with SES (Appendix: Table 11), and we found a flat relationship for High SES individuals and some suggestive evidence of an increase in mortality with cold temperatures for Low and Medium SES individuals. However, the size of the coefficients is small, and results are not statistically significant at the 95% level.^9^ Consequently, we do not observe any substantive effects of extreme temperatures in the age group 25–64.

As further robustness checks, we replicated the results in Fig. 2 for the pooled sample controlling for three age groups (65–74;75–84;85+)^10^ using a dataset that comprises observations by age group, gender, province, month, and year (Appendix: Table 12). The results are comparable to the ones found in Fig. 2 for the pooled sample and show an increase in mortality at higher ages. Moreover, we inquired the interaction between the three age categories and the temperature bins (Appendix: Table 13) finding the largest effect size in the age group 85+. We run a placebo test using the temperature bins from 5 months before the actual deaths finding small coefficients with limited statistical power substantiating our mains results (Appendix: Table 14). We tested an alternative modeling strategy running an OLS regression and using as outcome the mortality rate. The results showed to replicate the main analysis in Fig. 2 (Appendix: Table 15) and the results in Fig. 3 (Appendix: Table 16).^11^

## Discussion and Conclusion

In this article, we have inquired the educational gradient in temperature-related deaths for individuals aged above 65 in Spain from 2012 to 2018 and how it varies depending on the local climate. The article improves on previous research bringing further evidence of a stratified effect of temperature by SES using 50 Spanish Provinces compared to previous studies that focused only on Barcelona (Borrell et al., 2006; Marí-Dell’Olmo et al., 2019). Moreover, we exposed, for the first time, that the local climate shapes adaptation to heat and cold but mostly for low SES individuals.

The findings on the SES gradient are in line with the evidence on the higher risk of extreme temperatures for Low SES individuals. For example, analysis of the impact of the 2003 heatwave and extreme temperatures in Barcelona showed low SES individuals to be the most vulnerable (Borrell et al., 2006; Marí-Dell’Olmo et al., 2019). Likely, Low SES individuals are likely to be more exposed, less resilient and lack support in case of need when affronting a day with extreme temperatures (Gronlund, 2014; Hsu et al., 2021; Kovats & Hajat, 2008). For example, our findings on the protective role of marital status substantiate the higher vulnerability of Low and Medium SES individuals as they show to be more dependent on the support of a partner when exposed to extreme temperatures. Overall, we can describe the relationship between extreme temperature and mortality as U-shaped for Low and Medium SES individuals and flatter for High SES individuals.

For the first time, we exposed the local climate to moderate the effect of extreme heat in Spain and only for low SES individuals. Previous studies showed a larger effect of hot weather at higher latitudes or colder climates (Curriero et al., 2002; De’ Donato et al., 2015; Xiao et al., 2015). Here, we find the same pattern to be true as in the pooled sample we observe a negative coefficient when we interact days at the 95th percentile with the median temperature. The results suggest adaptation and acclimatization to sweltering days in warmer locations that was, partially, already documented in the long-term analysis of temperature-related deaths (Díaz et al., 2018). Moreover, we stratified the analysis by SES, and found statistically significant and sizeable effects only for low SES individuals when considering exposure to hot days and for low SES and Medium SES when looking at cold days. Possibly, the introduction of public policies could explain the reduction in the burden of hot days for low SES individuals in the warmer climates of Spain. For example, the “Heat Health Prevention Plan” implemented after the 2003 heatwave helped to prevent several deaths in the following years, especially in the Southern provinces of Spain (Martínez-Solanas & Basagaña, 2019).

The study has three main limitations. First, we cannot directly test the mechanisms that explain the SES gradient and the heterogeneous effect dependent on the climate. For example, in the dataset there is no relevant information on individual’s pre-existing morbidities or the housing conditions that could explain the educational gradient found in the analysis. Secondly, the administrative unit of analysis, Spanish Provinces, could be considered as large and determining some measurement error in the exposure to temperature. However, limitations in data availability do not allow us to collect population estimates and death counts at a more fine-grained spatial level. In previous studies, this issue has been addressed using average temperatures within an administrative unit weighted by the population living in the lower administrative units. For example, studies in the USA used temperature and mortality data at the state level and computed average temperatures weighted by the populations in the counties (Barreca et al., 2016). Here, we adopted the solution used by two studies that used the same unit of analysis, provinces in Spain, and relied on the average value of the grids falling within each province (Achebak et al., 2019, 2020). Based on previous studies, the use of more fine-grained units of analysis would not have affected the association we observed but could have determined a larger effect size (Clemens et al., 2021; Lee et al., 2016). Consequently, our study could be considered as showing a lower bound of the association between temperature and mortality in Spain. Thirdly, the dataset used is limited in its time span as information on educational attainment in Spain is not present before 2012. A larger time frame could have allowed us to observe how the educational gradient in heat-related deaths evolved over time. Similarly, it would have allowed us to explore how the “Heat Health Prevention Plan” introduced in 2003 affected vulnerability to heat in different educational groups.

Future research could tackle two main shortcomings of the current study. First, the mechanisms explaining the stratified effect of temperature on mortality could be explored to inform which public policies are the most effective to reduce risk of the most vulnerable social groups. For example, an analysis at a more precise geographical level could uncover disparities in exposure and provide evidence of targeted support for the most vulnerable that might be inhabiting houses settled in heat islands. Secondly, a study using a longer time frame could allow to investigate how adaptation occurred for different SES groups detecting critical turning points in the SES gradient in heat-related deaths.

In conclusion, climate change poses new risks of public health that policymakers should address to protect vulnerable individuals, such as the low educated individuals aged above 65 residing in colder climates, and protect them from new unexpected meteorological threats affecting their wellbeing and survival.

## Data Availability

The datasets generated during and/or analyzed during the current study are not publicly available as the INE dataset on mortality has restricted access but can be requested to the competent authorities. Conversely, the other datasets used in the analysis are all publicly accessible.

## Appendix

**Table 2:** INE and EPA estimates of the percentage of individuals above 16 by SES group

Variable	Obs.	Mean	Std. Dev.	Min	Max
*Low SES*					
INE	85	7.24	3.73	1.55	14.96
EPA	85	7.75	4.16	1.47	16.54
*Medium SES*					
INE	85	15.05	3.18	9.37	23
EPA	85	15.89	3.36	10.03	24.35
High SES					
INE	85	77.72	4.01	71.45	85.55
EPA	85	76.36	4.3	69.39	84.98

The table provides summary statistics on the percentage of individuals by educational attainment resident in 17 regions in Spain between 2014 and 2018 based on INE and EPA data

See Fig. ^6^ and Tables 2,

**Table 3:** Extreme temperature & mortality

	(1)
≤ 5th percentile	0.007***
	(0.001)
> 5th and ≤ 10th percentile	0.003**
	(0.001)
> 10th and ≤ 25th percentile	0.003***
	(0.001)
≥ 75th and < 90th percentile	−0.001
	(0.001)
≥ 90th and < 95th percentile	0.000
	(0.001)
≥ 95th percentile	0.005***
	(0.001)
Medium SES	−0.197***
	(0.047)
High SES	−0.281***
	(0.053)
Female	−0.187***
	(0.007)
Log GDP per capita	0.027
	(0.075)
Log PM 2.5	0.008
	(0.006)
Population density	−0.000*
	(0.000)
Solar Radiation	−0.001
	(0.001)
Wind speed	−0.000
	(0.002)
Precipitation	−0.002
	(0.001)
Relative humidity	−0.001***
	(0.000)
Observations	25,200
Province-Month FE	YES
Month-Year FE	YES

Results are obtained estimating Eq. (1) for the pooled sample. Standard errors are clustered at the provincial level. Constant present but not reported. Significance levels: ****p* < 0.001, ***p* < 0.01, **p* < 0.05

^3^,

**Table 4:** Extreme temperature, mortality & interaction with SES

	(1) Interaction with SES
≤ 5th percentile	0.001
	(0.002)
≤ 5th percentile # Low SES	0.007***
	(0.001)
≤ 5th percentile # Medium SES	0.007***
	(0.002)
> 5th and ≤ 10th percentile	0.010
	(0.005)
> 5th and ≤ 10th percentile # Low SES	−0.008***
	(0.002)
> 5th and ≤ 10th percentile # Medium SES	−0.013**
	(0.004)
> 10th and ≤ 25th percentile	0.001
	(0.001)
> 10th and ≤ 25th percentile # Low SES	0.002*
	(0.001)
> 10th and ≤ 25th percentile # Medium SES	0.001
	(0.001)
≥ 75th and < 90th percentile	0.004
	(0.003)
≥ 75th and < 90th percentile # Low SES	−0.005***
	(0.001)
≥ 75th and < 90th percentile # Medium SES	−0.006***
	(0.001)
≥ 90th and < 95th percentile	−0.000
	(0.001)
≥ 90th and < 95th percentile # Low SES	0.000
	(0.002)
≥ 90th and < 95th percentile # Medium SES	0.003
	(0.002)
≥ 95th percentile	0.002
	(0.002)
≥ 95th percentile # Low SES	0.006***
	(0.001)
≥ 95th percentile # Medium SES	0.006***
	(0.001)
Observations	25,200
Controls	YES
Province-Month FE	YES
Month-Year FE	YES

Results are obtained estimating Eq. (1) adding an interaction between the temperature bins and SES groups. The High SES group is at the baseline level. Standard errors are clustered at the provincial level. Constant present but not reported. Control variables are precipitation, solar radiation, wind speed, relative humidity, GDP per capita, PM2.5, gender and population density. Significance levels: ****p* < 0.001, ***p* < 0.01, **p* < 0.05

^4^,

**Table 5:** Temperature-related deaths & interaction with median temperature

	(1) Pooled
≤ 5th percentile	−0.001
	(0.004)
≤ 5th percentile # Median Temperature	0.001*
	(0.000)
> 5th and ≤ 10th percentile	0.002
	(0.005)
> 5th and ≤ 10th percentile # Median Temperature	0.000
	(0.000)
> 10th and ≤ 25th percentile	0.003
	(0.003)
> 10th and ≤ 25th percentile # Median Temperature	0.000
	(0.000)
≥ 75th and < 90th percentile	−0.003
	(0.002)
≥ 75th and < 90th percentile # Median Temperature	0.000
	(0.000)
≥ 90th and < 95th percentile	−0.004
	(0.005)
≥ 90th and < 95th percentile # Median Temperature	0.000
	(0.000)
≥ 95th percentile	0.013**
	(0.004)
≥ 95th percentile # Median Temperature	−0.001*
	(0.000)
Observations	25,200
Controls	YES
Province-Month FE	YES
Month-Year FE	YES

Results are obtained estimating Eq. (1) adding an interaction between the temperature bins and the median temperature of the province. Standard errors are clustered at the provincial level. Constant present but not reported. Control variables are precipitation, solar radiation, wind speed, relative humidity, GDP per capita, PM2.5, gender, SES and population density. Significance levels: ****p* < 0.001, ***p* < 0.01, **p* < 0.05

^5^,

**Table 6:** Temperature-related deaths & interaction with SES groups and median temperature

	(1)
≤ 5th percentile	−0.003
	(0.007)
≤ 5th percentile # Medium SES	0.004
	(0.008)
≤ 5th percentile # High SES	−0.003
	(0.008)
≤ 5th percentile # Median Temperature	0.001
	(0.000)
≤ 5th percentile # Medium SES # Median Temperature	−0.000
	(0.000)
≤ 5th percentile # High SES # Median Temperature	−0.000
	(0.000)
> 5th and ≤ 10th percentile	0.004
	(0.010)
> 5th and ≤ 10th percentile # Medium SES	−0.006
	(0.016)
> 5th and ≤ 10th percentile # High SES	0.000
	(0.013)
> 5th and ≤ 10th percentile # Median Temperature	−0.000
	(0.001)
> 5th and ≤ 10th percentile # Medium SES # Median Temperature	0.000
	(0.001)
> 5th and ≤ 10th percentile # High SES # Median Temperature	0.000
	(0.001)
≥ 90th and < 95th percentile	−0.022**
	(0.008)
≥ 90th and < 95th percentile # Medium SES	0.022*
	(0.011)
≥ 90th and < 95th percentile # High SES	0.023
	(0.010)
≥ 90th and < 95th percentile # Median Temperature	0.001*
	(0.001)
≥ 90th and < 95th percentile # Medium SES # Median Temperature	−0.001
	(0.001)
≥ 90th and < 95th percentile # High SES # Median Temperature	−0.001
	(0.001)
≥ 95th percentile	0.034***
	(0.007)
≥ 95th percentile # Medium SES	−0.023**
	(0.007)
≥ 95th percentile # High SES	−0.030***
	(0.009)
≥ 95th percentile # Median Temperature	−0.002***
	(0.000)
≥ 95th percentile # Medium SES # Median Temperature	0.002**
	(0.000)
≥ 95th percentile # High SES # Median Temperature	0.002*
	(0.001)
Observations	25,200
Controls	YES
Province-Month FE	YES
Month-Year FE	YES

Results are obtained estimating Eq. (1) adding a three-way interaction between the temperature bins, the median temperature of the province and the SES groups. For conciseness, the categories with temperature between the 10th and 90th percentile are not included and considered as the comfort zone. Constant present but not reported. The Low SES category is at the reference level. Control variables are precipitation, solar radiation, wind speed, relative humidity, GDP per capita, PM2.5, gender and population density. Standard errors are clustered at the provincial level. Significance levels: ****p* < 0.001, ***p* < 0.01, **p* < 0.05

^6^,

**Table 7:** Temperature-related deaths and interaction with gender

Variables	(1)
≤ 5th percentile	0.005***
	(0.001)
≤ 5th percentile*Female	0.003***
	(0.001)
> 5th and ≤ 10th percentile	0.002
	(0.001)
> 5th and ≤ 10th percentile*Female	0.001
	(0.001)
> 10th and ≤ 25th percentile	0.002*
	(0.001)
> 10th and ≤ 25th percentile*Female	0.001**
	(0.000)
≥ 75th and < 90th percentile	0.000
	(0.001)
≥ 75th and < 90th percentile* Female	−0.001*
	(0.000)
≥ 90th and < 95th percentile	−0.001
	(0.001)
≥ 90th and < 95th percentile*Female	0.002***
	(0.001)
≥ 95th percentile	0.004***
	(0.001)
≥ 95th percentile*Female	0.002***
	(0.000)
Observations	25,200
Province-Month FE	YES
Month-Year FE	YES

Results are obtained estimating Eq. (1) adding an interaction with gender. Male is the baseline category. Standard errors are clustered at the provincial level. Constant present but not reported. Control variables are precipitation, solar radiation, wind speed, relative humidity, GDP per capita, PM2.5, SES and population density. Significance levels: ****p* < 0.001, ***p* < 0.01, **p* < 0.05

^7^,

**Table 8:** Temperature-related deaths and interaction with gender by SES groups

Variables	(1)	(2)	(3)
	Low SES	Medium SES	High SES
≤ 5th percentile	0.006*	0.005**	0.003
	(0.002)	(0.002)	(0.003)
≤ 5th percentile*Female	0.003***	0.002	0.002
	(0.001)	(0.001)	(0.001)
> 5th and ≤ 10th percentile	0.005*	0.004	−0.001
	(0.003)	(0.002)	(0.002)
> 5th and ≤ 10th percentile*Female	−0.000	−0.001	0.003
	(0.002)	(0.001)	(0.001)
> 10th and ≤ 25th percentile	0.003*	−0.001	0.002
	(0.001)	(0.001)	(0.002)
> 10th and ≤ 25th percentile*Female	0.002**	0.001	0.001*
	(0.001)	(0.001)	(0.001)
≥ 75th and < 90th percentile	0.001	−0.000	−0.002
	(0.001)	(0.001)	(0.001)
≥ 75th and < 90th percentile* Female	−0.000	−0.001**	−0.000
	(0.001)	(0.001)	(0.000)
≥ 90th and < 95th percentile	−0.001	−0.001	−0.003
	(0.002)	(0.001)	(0.002)
≥ 90th and < 95th percentile*Female	0.004**	0.004***	0.004**
	(0.001)	(0.001)	(0.001)
≥ 95th percentile	0.005**	0.004***	0.004*
	(0.002)	(0.001)	(0.002)
≥ 95th percentile*Female	0.002*	0.001*	−0.001
	(0.001)	(0.001)	(0.001)
Observations	8,400	8,400	8,400
Province-Month FE	YES	YES	YES
Month-Year FE	YES	YES	YES

Results are obtained estimating Eq. (1) separately by SES group adding an interaction with gender. Male is the baseline category. Standard errors are clustered at the provincial level. Constant present but not reported. Control variables are precipitation, solar radiation, wind speed, relative humidity, GDP per capita, PM2.5, and population density. Significance levels: ****p* < 0.001, ***p* < 0.01, **p* < 0.05

^8^,

**Table 9:** Temperature-related deaths and interaction with marriage status

	(1)
≤ 5th percentile	0.009***
	(0.002)
≤ 5th percentile*Married	−0.005***
	(0.001)
> 5th and ≤ 10th percentile	0.002*
	(0.001)
> 5th and ≤ 10th percentile*Married	0.001
	(0.001)
> 10th and ≤ 25th percentile	0.003*
	(0.001)
> 10th and ≤ 25th percentile*Married	−0.003***
	(0.000)
≥ 75th and < 90th percentile	−0.001
	(0.001)
≥ 75th and < 90th percentile*Married	0.002***
	(0.000)
≥ 90th and < 95th percentile	0.001***
	(0.000)
≥ 90th and < 95th percentile*Married	−0.001*
	(0.001)
≥ 95th percentile	0.006***
	(0.001)
≥ 95th percentile*Married	−0.002***
	(0.000)
Observations	50,400
Province-Month FE	YES
Month-Year FE	YES

Results are obtained estimating Eq. (1) adding an interaction with marital status. Standard errors are clustered at the provincial level. Constant present but not reported. Control variables are precipitation, solar radiation, wind speed, relative humidity, GDP per capita, PM2.5, gender, SES and population density. Significance levels: ****p* < 0.001, ***p* < 0.01, **p* < 0.05

^9^,

**Table 10:** Temperature-related deaths by SES group and interaction with marriage status

	(1)	(2)	(3)
	Low SES	Middle SES	High SES
≤ 5th percentile	0.008***	0.009***	0.005
	(0.002)	(0.002)	(0.003)
≤ 5th percentile*Married	−0.005***	−0.005***	−0.003***
	(0.001)	(0.001)	(0.001)
> 5th and ≤ 10th percentile	0.004*	0.002	−0.000
	(0.002)	(0.002)	(0.002)
> 5th and ≤ 10th percentile*Married	0.003	0.003	0.000
	(0.001)	(0.002)	(0.001)
> 10th and ≤ 25th percentile	0.004***	0.002	0.004*
	(0.001)	(0.001)	(0.002)
> 10th and ≤ 25th percentile*Married	−0.003***	−0.003**	−0.003***
	(0.001)	(0.001)	(0.001)
≥ 75th and < 90th percentile	−0.000	−0.002	−0.002
	(0.001)	(0.001)	(0.002)
≥ 75th and < 90th percentile*Married	0.002***	0.002***	0.003***
	(0.001)	(0.000)	(0.000)
≥ 90th and < 95th percentile	0.002	0.002	0.001
	(0.002)	(0.002)	(0.002)
≥ 90th and < 95th percentile*Married	−0.004*	−0.001	−0.004***
	(0.002)	(0.001)	(0.001)
≥ 95th percentile	0.007***	0.007***	0.004*
	(0.002)	(0.001)	(0.002)
≥ 95th percentile*Married	−0.001	−0.002**	−0.000
	(0.001)	(0.001)	(0.001)
Observations	16,800	16,800	16,800
Province-Month FE	YES	YES	YES
Month-Year FE	YES	YES	YES

Results are obtained estimating Eq. (1) separately by SES group and adding an interaction with marital status. Standard errors are clustered at the provincial level. Constant present but not reported. Control variables are precipitation, solar radiation, wind speed, relative humidity, GDP per capita, PM2.5, gender and population density. Significance levels: ****p* < 0.001, ***p* < 0.01, **p* < 0.05

^10^,

**Table 11:** Temperature-related deaths for individuals aged below 65 and interaction with SES

	(1)
≤ 5th percentile	−0.16
	(0.019)
≤ 5th percentile*Low SES	0.021
	(0.011)
≤ 5th percentile*Medium SES	0.025**
	(0.009)
> 5th and ≤ 10th percentile	0.028
	(0.018)
> 5th and ≤ 10th percentile*Low SES	0.002
	(0.018)
> 5th and ≤ 10th percentile*Medium SES	−0.040**
	(0.015)
> 10th and ≤ 25th percentile	0.09
	(0.012)
> 10th and ≤ 25th percentile*Low SES	0.007
	(0.007)
> 10th and ≤ 25th percentile*Medium SES	0.019*
	(0.010)
≥ 75th and < 90th percentile	0.005
	(0.010)
≥ 75th and < 90th percentile*Low SES	−0.020
	(0.011)
≥ 75th and < 90th percentile*Medium SES	−0.002
	(0.008)
≥ 90th and < 95th percentile	0.008
	(0.011)
≥ 90th and < 95th percentile*Low SES	−0.020
	(0.017)
≥ 90th and < 95th percentile*Medium SES	−0.002
	(0.016)
≥ 95th percentile	−0.017
	(0.013)
≥ 95th percentile*Low SES	−0.002
	(0.013)
≥ 95th percentile*Medium SES	−0.006
	(0.011)
Observations	18,216
Province-Month FE	YES
Month-Year FE	YES

Results are obtained estimating Eq. (1) adding an interaction with SES. Standard errors are clustered at the provincial level. Significance levels: Constant present but not reported. Control variables are precipitation, solar radiation, wind speed, relative humidity, GDP per capita, PM2.5, gender and population density. ****p* < 0.001, ***p* < 0.01, **p* < 0.05

^11^,

**Table 12:** Temperature-related deaths controlling for age categories

	(1)
≤ 5th percentile	0.006***
	(0.001)
> 5th and ≤ 10th percentile	0.002
	(0.001)
> 10th and ≤ 25th percentile	0.002
	(0.001)
≥ 75th and < 90th percentile	−0.000
	(0.001)
≥ 90th and < 95th percentile	0.000
	(0.001)
≥ 95th percentile	0.005***
	(0.001)
Age category 75 to 84	1.131***
	(0.005)
Age category 85+	2.380***
	(0.010)
Observations	25,200
Controls	YES
Province-Month FE	YES
Month-Year FE	YES

Results are obtained estimating Eq. (1) adding age categories as control variables but no controls for educational groups for lack of data. Standard errors are clustered at the provincial level. Constant present but not reported. Control variables are precipitation, solar radiation, wind speed, relative humidity, GDP per capita, PM2.5, gender and population density. Significance levels: ****p* < 0.001, ***p* < 0.01, **p* < 0.05

^12^,

**Table 13:** Temperature-related deaths and interaction with age categories

	(1)
≤ 5th percentile	−0.002*
	(0.001)
≤ 5th percentile* Age category 75 to 84	0.006***
	(0.001)
≤ 5th percentile* Age category 85+	0.011***
	(0.001)
> 5th and ≤ 10th percentile	0.001
	(0.001)
> 5th and ≤ 10th percentile* Age category 75 to 84	0.003***
	(0.000)
> 5th and ≤ 10th percentile * Age category 85+	0.001
	(0.001)
> 10th and ≤ 25th percentile	−0.000
	(0.001)
> 10th and ≤ 25th percentile* Age category 75 to 84	0.001**
	(0.000)
> 10th and ≤ 25th percentile* Age category 85+	0.004***
	(0.000)
≥ 75th and < 90th percentile	0.002*
	(0.001)
≥ 75th and < 90th percentile* Age category 75 to 84	−0.002***
	(0.000)
≥ 75th and < 90th percentile* Age category 85+	−0.003***
	(0.000)
≥ 90th and < 95th percentile	0.001
	(0.001)
≥ 90th and < 95th percentile* Age category 75 to 84	−0.001
	(0.001)
≥ 90th and < 95th percentile* Age category 85+	0.001
	(0.001)
> 95th percentile	0.004***
	(0.001)
> 95th percentile* Age category 75 to 84	0.001
	(0.000)
> 95th percentile* Age category 85+	0.002***
	(0.001)
Observations	25,200
Province-Month FE	YES
Month-Year FE	YES

Results are obtained estimating Eq. (1) adding an interaction with age categories. Standard errors are clustered at the provincial level. Constant present but not reported. Control variables are precipitation, solar radiation, wind speed, relative humidity, GDP per capita, PM2.5, gender and population density. Significance levels: ****p* < 0.001, ***p* < 0.01, **p* < 0.05

^13^,

**Table 14:** Placebo test

	(1) Placebo
Lag ≤ 5th	0.000
	(0.001)
Lag > 5th and ≤ 10th percentile	−0.001
	(0.001)
Lag > 10th and ≤ 25th percentile	0.000
	(0.001)
Lag ≥ 75th and < 90th percentile	0.000
	(0.001)
Lag ≥ 90th and < 95th percentile	0.000
	(0.001)
Lag ≥ 95th	0.000
	(0.001)
Observations	25,200
Province-Month FE	YES
Month-Year FE	YES

Results are obtained estimating Eq. (1) for the pooled sample and temperatures five months before. Standard errors clustered at the provincial level. Constant present but not reported. Control variables are precipitation, solar radiation, wind speed, relative humidity, GDP per capita, PM2.5, gender, SES and population density. Significance levels: ****p* < 0.001, ***p* < 0.01, **p* < 0.05

^14^,

**Table 15:** Extreme temperature and mortality rate with OLS regression

	(1) OLS
≤ 5th percentile	0.320***
	(0.070)
> 5th and ≤ 10th percentile	0.092
	(0.070)
> 10th and ≤ 25th percentile	0.115*
	(0.045)
≥ 75th and < 90th percentile	−0.049
	(0.033)
≥ 90th and < 95th percentile	0.03
	(0.052)
≥ 95th percentile	0.228***
	(0.051)
Observations	25,200
Province-Month FE	YES
Month-Year FE	YES

Results are obtained estimating an OLS regression on the mortality rate with month-year and province-month FE and the control variables used in Eq. (1) for individuals aged above 65. Constant present but not reported. Control variables are precipitation, solar radiation, wind speed, relative humidity, GDP per capita, PM2.5, gender, SES and population density Standard errors clustered at the provincial level. Significance levels: ****p* < 0.001, ***p* < 0.01, **p* < 0.05

^15^ and

**Table 16:** Extreme temperature, mortality rate with OLS regression & interaction with SES

	(1) Interaction with SES
≤ 5th percentile	0.011
	(0.099)
≤ 5th percentile # Low SES	0.57***
	(0.094)
≤ 5th percentile # Medium SES	0.259*
	(0.109)
> 5th and ≤ 10th percentile	0.402**
	(0.123)
> 5th and ≤ 10th percentile # Low SES	−0.474**
	(0.170)
> 5th and ≤ 10th percentile # Medium SES	−0.533**
	(0.176)
> 10th and ≤ 25th percentile	0.001
	(0.064)
> 10th and ≤ 25th percentile # Low SES	0.219***
	(0.058)
> 10th and ≤ 25th percentile # Medium SES	0.046
	(0.060)
≥ 75th and < 90th percentile	0.185***
	(0.045)
≥ 75th and < 90th percentile # Low SES	−0.309***
	(0.045)
≥ 75th and < 90th percentile # Medium SES	−0.292***
	(0.039)
≥ 90th and < 95th percentile	−0.012
	(0.069)
≥ 90th and < 95th percentile # Low SES	0.110
	(0.103)
≥ 90th and < 95th percentile # Medium SES	0.111
	(0.100)
≥ 95th percentile	0.044
	(0.055)
≥ 95th percentile # Low SES	0.368***
	(0.071)
≥ 95th percentile # Medium SES	0.319***
	(0.057)
Observations	25,200
Province-Month FE	YES
Month-Year FE	YES

Results are obtained estimating an OLS regression on the mortality rate with month-year and province-month FE, an interaction with the three SES groups and adding the control variables used in Eq. (1) for individuals aged above 65. Constant present but not reported. Control variables are precipitation, solar radiation, wind speed, relative humidity, GDP per capita, PM2.5, gender, SES and population density Standard errors clustered at the provincial level. Significance levels: ****p* < 0.001, ***p* < 0.01, **p* < 0.05

^16^.

## Footnotes

1 In this paper, we consider all provinces except for Ceuta and Melilla that are in the northern part of Morocco and for which we have missing information for the environmental variables of interest.

2 In the remaining of the paper, we will use SES and educational attainment interchangeably.

3 Due to data availability, studies have mostly relied on the educational attainment of individuals to evaluate how SES stratifies the effect of temperature on mortality. Some studies have used occupation, but using this indicator would not allow to effectively classify the SES of retired individuals. Moreover, other studies inquired how the neighborhood SES stratifies the effect of temperature, finding a stronger relationship in low SES neighborhoods (Madrigano et al., 2015).

4 The deceased individuals over 65 from 2012 to 2018 are 32.6% Low SES, 37% Medium SES and 30.4% High SES.

5 The dataset misses information on the educational attainment of 1.5% of the total.

6 More information on the data provided by the Spanish Ministry of Ecological Transition can be found here: https://www.miteco.gob.es/es/calidad-y-evaluacion-ambiental/temas/atmosfera-y-calidad-del-aire/calidad-del-aire/visor/default.aspx.

7 The average population of Spanish provinces is of about 932,736 inhabitants between 2012 and 2018. The least inhabited province is Soria with 88,600 inhabitants in 2018. The most inhabited province is Madrid with 6,578,079 residents.

8 The average size of a province is of 10,118km^2^ and Badajoz is the largest with 21,767 km^2^ and Guipuzcoa the smallest with 1,998 km.^2.^

9 There are some caveats to this analysis. First, the sample of deaths in this age group during these years is limited to 3,837. Secondly, we use the same SES groups used in the main analysis that might be less fitting for this age group but allows us to use a comparable measure of SES.

10 We cannot stratify the analysis by education and age groups above the age of 65 (e.g.: for individuals aged 65–75, 75–85 and 85 +) as the EPA survey provides information on the individuals aged above 65 in one age category.

11 In the analysis reported in Appendix: Table 14 and Appendix: Table 15, we did not include population weights. However, when including population weights for the month-years of exposure or for the total population of the provinces, we replicated similar findings to the ones presented. These results are available upon request.
